# Supplementary material for: Podocyte OTUD5 alleviates diabetic kidney disease through deubiquitinating TAK1 and reducing podocyte inflammation and injury
Source: Nat Commun. 2024 Jun 27;15:5441. doi: 10.1038/s41467-024-49854-1 (PMC11211476; doi:10.1038/s41467-024-49854-1)
Supplement: Supplementary file 1 — Supplementary Information [file 41467_2024_49854_MOESM1_ESM.pdf]

Supplementary Information

Podocyte OTUD5 alleviates diabetic kidney disease through deubiquitinating TAK1 and reducing podocyte inflammation and injury

The supplementary file includes 2 Table and 7 Figures.

Supplementary Table 1. Primer sequences for RT-qPCR assay.

Gene	Species	Sequence
Il6	Mouse	CTCCCAACAGACCTGTCTATAC
		CCATTGCACAACTCTTTTCTCA
Tnfα	Mouse	ATGTCTCAGCCTCTTCTCATTC
		GCTTGTCACTCGAATTTTGAGA
Otud5	Mouse	CTGCCTTTGGTCTGAATGATTG
		GTCTAGGTATTCCTGTTGGGAC
β-actin	Mouse	CTACCTCATGAAGATCCTGACC
		CACAGCTTCTCTTTGATGTCAC

Supplementary Table S2. Clinical data from human subjects with or without DKD.

Patient	Age	Sex	Scr (μmol/L)	eGFR (mL/min/1.73m^2)	UPE/24h (g)	FBG (mmol/L)
Normal group						
1	55-60	F	84	114.7	<0.15	6.2
2	75-80	M	61	82.4	<0.15	5.1
3	70-75	M	59	97.9	<0.15	4.8
DKD group						
1	75-80	F	97	48	3.6	12.1
2	45-50	F	135	40.4	3.4	18.7
3	60-65	M	202	29.2	6.4	13.2

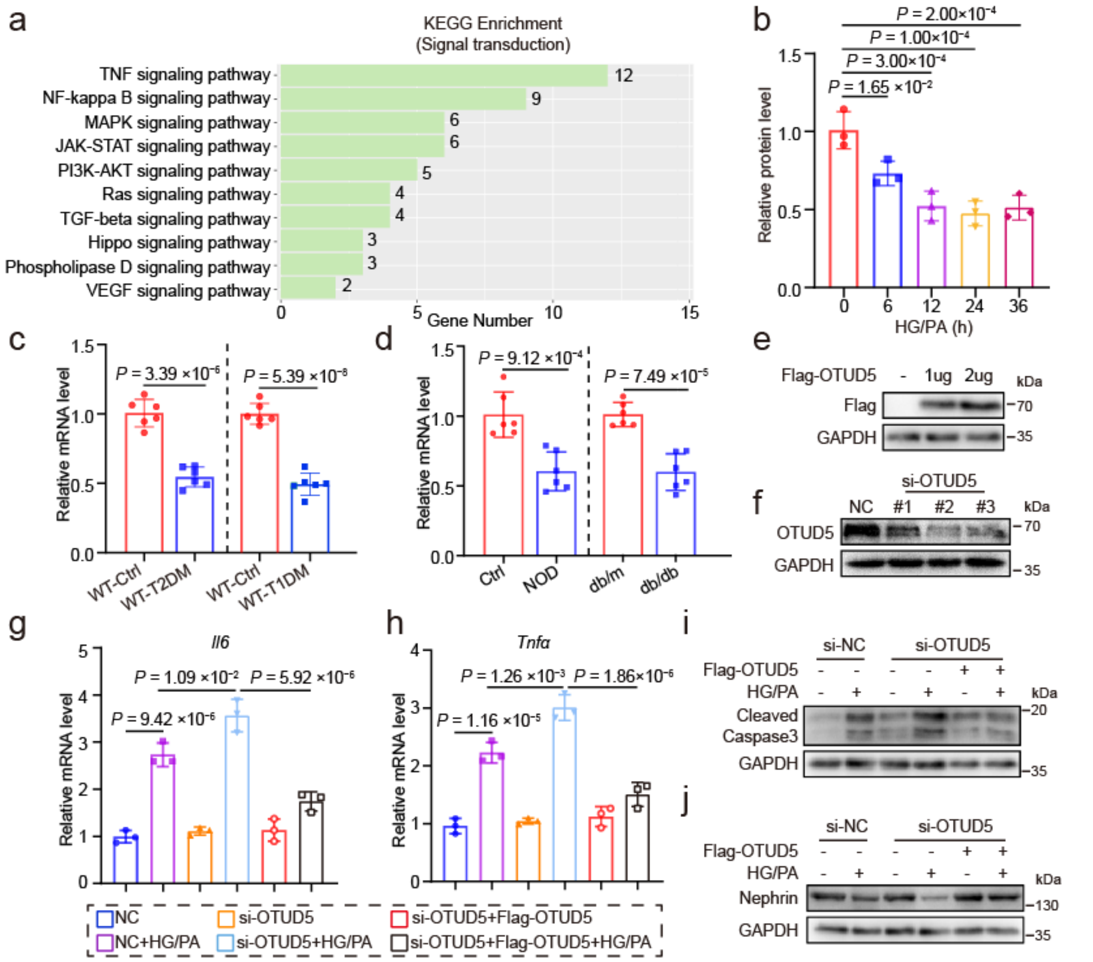

Supplementary Figure 1: Identification of OTUD5 as a regulator of podocyte inflammation and injury.

(a) KEGG enrichments of differentially expressed genes. (b) Densitometric quantification of the immunoblot in Figure 1e. ($n = 3$ independent experiments; P values were determined by one-way ANOVA with Bonferroni's correction). (c) Relative mRNA levels of *Otud5* in renal cortex of T2DM mice and T1DM mice. ($n = 6$ samples; P values were determined by Two-tailed unpaired t test). (d) Relative mRNA levels of *Otud5* in renal cortex of NOD mice and db/db mice. ($n = 6$ samples; P values were determined by two-tailed unpaired t test). (e) Representative western blot of Flag expression in podocytes transfected with gradient Flag-OTUD5. ($n = 3$ independent experiments). (f) Representative western blot of OTUD5 expression in podocytes transfected with different si-OTUD5 sequences. ($n = 3$ independent experiments). (g-h) Real-time qPCR analysis of *Il6* (g) and *Tnfα* (h) in OTUD5 knockdown MPC5 transfected with Flag-OTUD5 plasmid followed by HG/PA for 8h. ($n = 3$ independent experiments; P values were determined by one-way ANOVA with Bonferroni's correction.). (i-j) Representative western blot of Cleaved Caspase3 (i) and Nephrin (j) expression in OTUD5 knockdown MPC5 transfected with Flag-OTUD5 plasmid followed by HG/PA. ($n = 3$ independent experiments). For b, c, d, g and h, data are presented as mean ± SD.

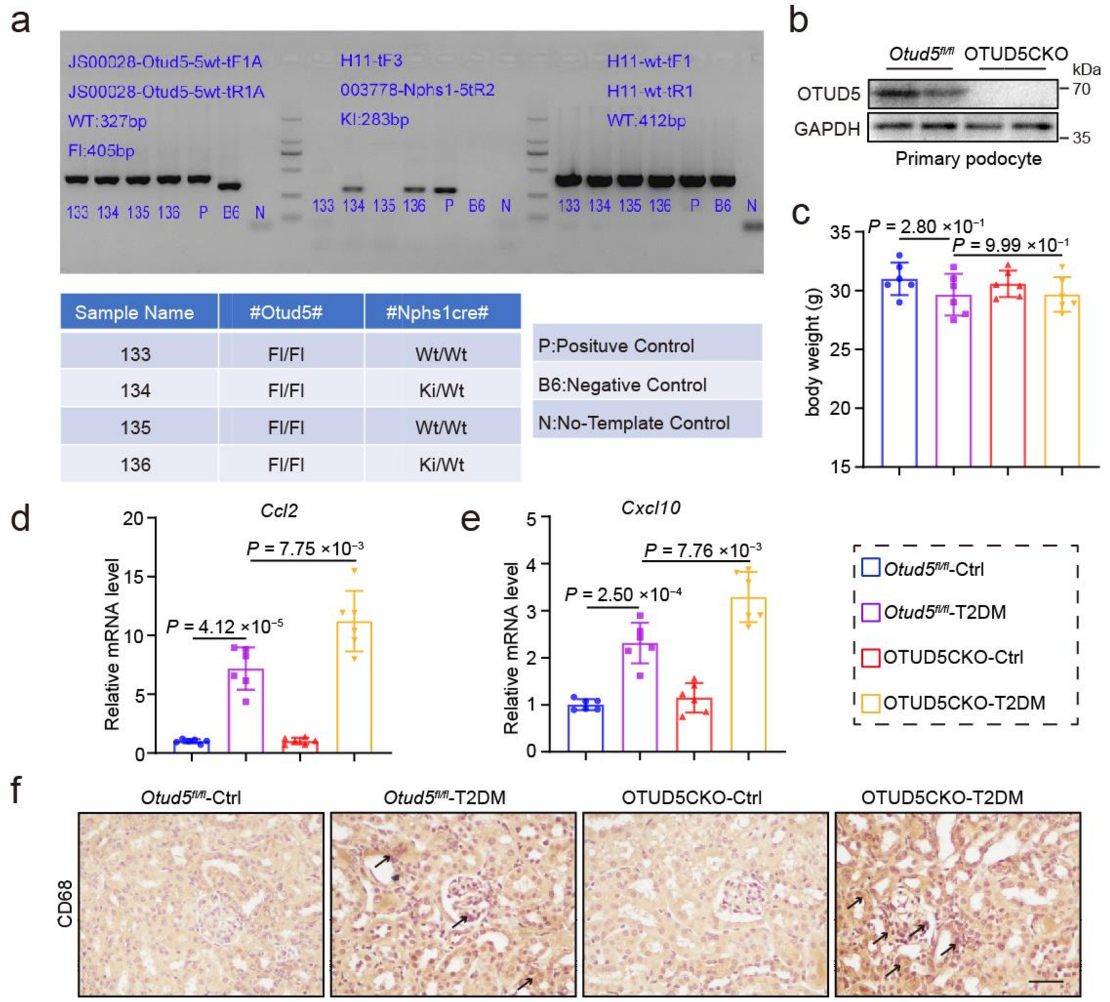

Supplementary Figure 2: Podocyte-specific *Otud5* knockout aggravates macrophage infiltration in T2DM mouse kidney.

(a) The primers of *Otud5* (WT:327bp, FI:405bp) and *Nphs-iCre* (KI:283bp) were respectively used for PCR to identify the genotype of mice. (b) Representative Western blot of OTUD5 expression in the kidney from *OTUD5*$^{fl/fl}$ and OTUD5CKO mice. ($n = 6$ samples). (c) The levels of body weight in mice.(n=6 samples; *P* values were determined by one-way ANOVA with Bonferroni's correction). (d-e) Real-time qPCR showing mRNA levels of *Ccl2* (e) and *Cxcl10* (f) in kidney tissues of each group. ($n = 6$ samples; *P* values were determined by one-way ANOVA with Bonferroni's correction. ns= no significance). (f) Representative immunohistochemistry (IHC) images of CD68 expression in mice. Scale bar, 50 μm. ($n = 6$ samples). For c-e, data are presented as mean ± SD.

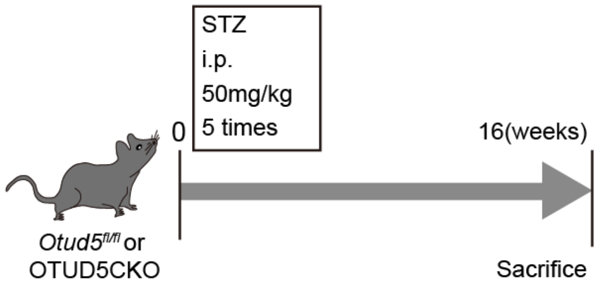

Supplementary Figure 3: Schematic diagram depicting the procedure of STZ-induced T1DM mice.

a

Gene names	Score
Ighg2b, Igkc	323.31
OTUD5	323.31
Myh9	323.31
Actb	313.54
Rps18	267.27
Myl12a;Myl12b	187.21
Rpl13	165.27
Igkv4-55	158.01
Ighg;Igh-1a	154.41
TAK1	146.78
Grn	144.6
Myl6	132.87
Rpl27	119.08
Myh10	94.822
Rps3	87.312

b

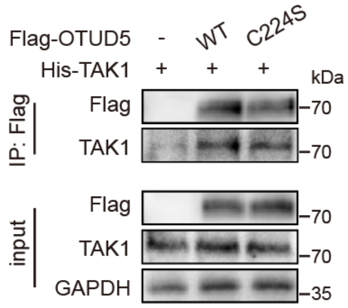

Supplementary Figure 4: Identification of TAK1 as a potential substrate protein of OTUD5.

(a) A table showing the candidate substrates of OTUD5 screened by the interactomes. (b) Co-IP of OTUD5 in NIH/3T3 co-transfected with Flag-OTUD5 (WT or C24A) and His-TAK1 plasmids. Exogenous OTUD5 was immunoprecipitated using anti-Flag antibody. (*n* = 3 independent experiments)

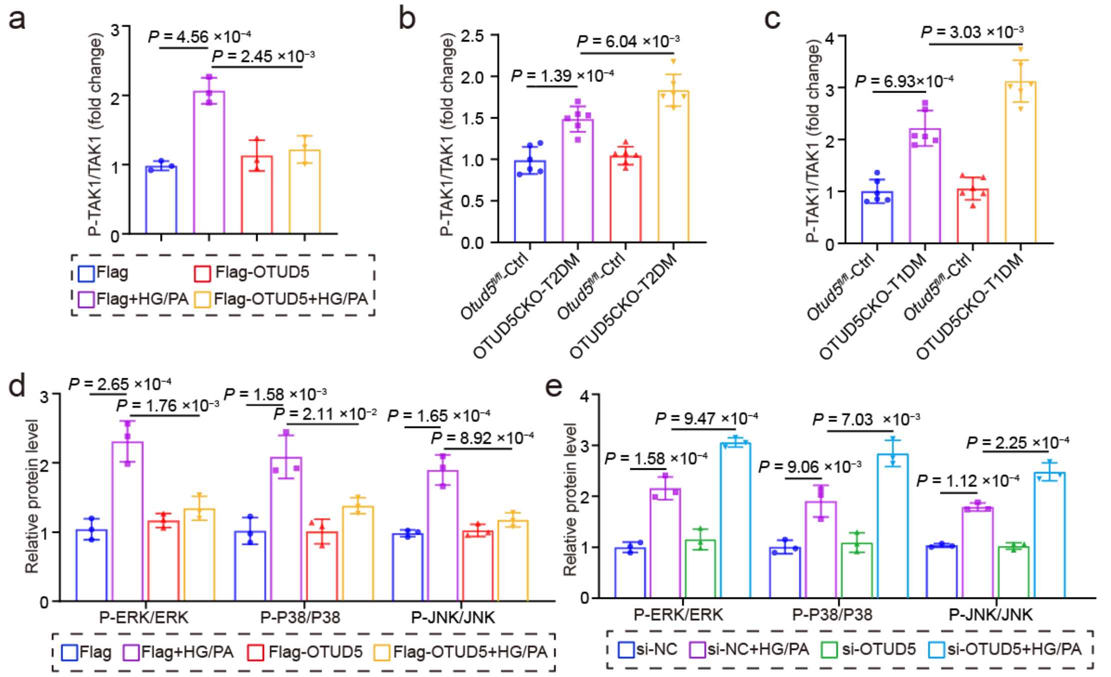

Supplementary Figure 5: OTUD5 negatively regulates TAK1-MAPK pathway activation in podocytes.

(a) Densitometric quantification of immunoblots in Figure 5a. ($n = 3$ independent experiments; P values were determined by one-way ANOVA with Bonferroni's correction). (b-c) Densitometric quantification of immunoblots in Figure 5c-d. ($n = 6$ samples; P values were determined by one-way ANOVA with Bonferroni's correction). (d-e) Densitometric quantification of immunoblots in Figure 5e-f. ($n = 3$ independent experiments; P values were determined by one-way ANOVA with Bonferroni's correction). For a-e, data are presented as mean ± SD.

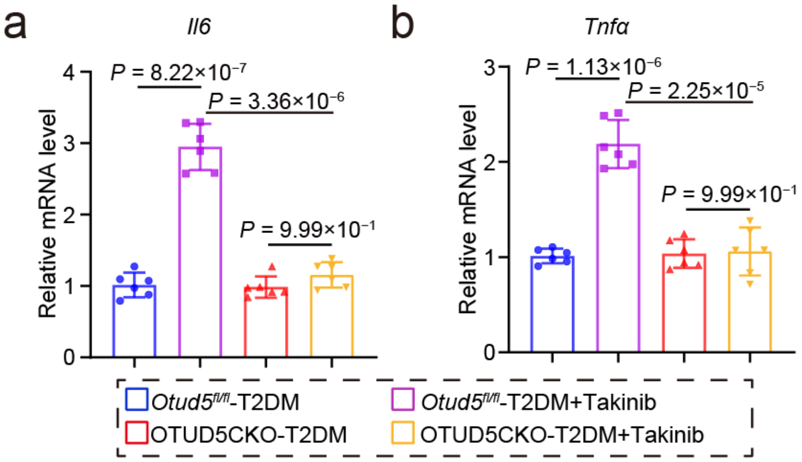

Supplementary Figure 6: Inhibition of TAK1 eliminates the aggravated podocyte inflammatory cytokines in OTUD5CKO-T2DM mice.

(a-b) Real-time qPCR showing mRNA levels of *Il6* (a) and *Tnfα* (b) in the kidney tissues of each group. ($n = 6$ samples; P values were determined by one-way ANOVA with Bonferroni's correction and data are presented as mean ± SD).

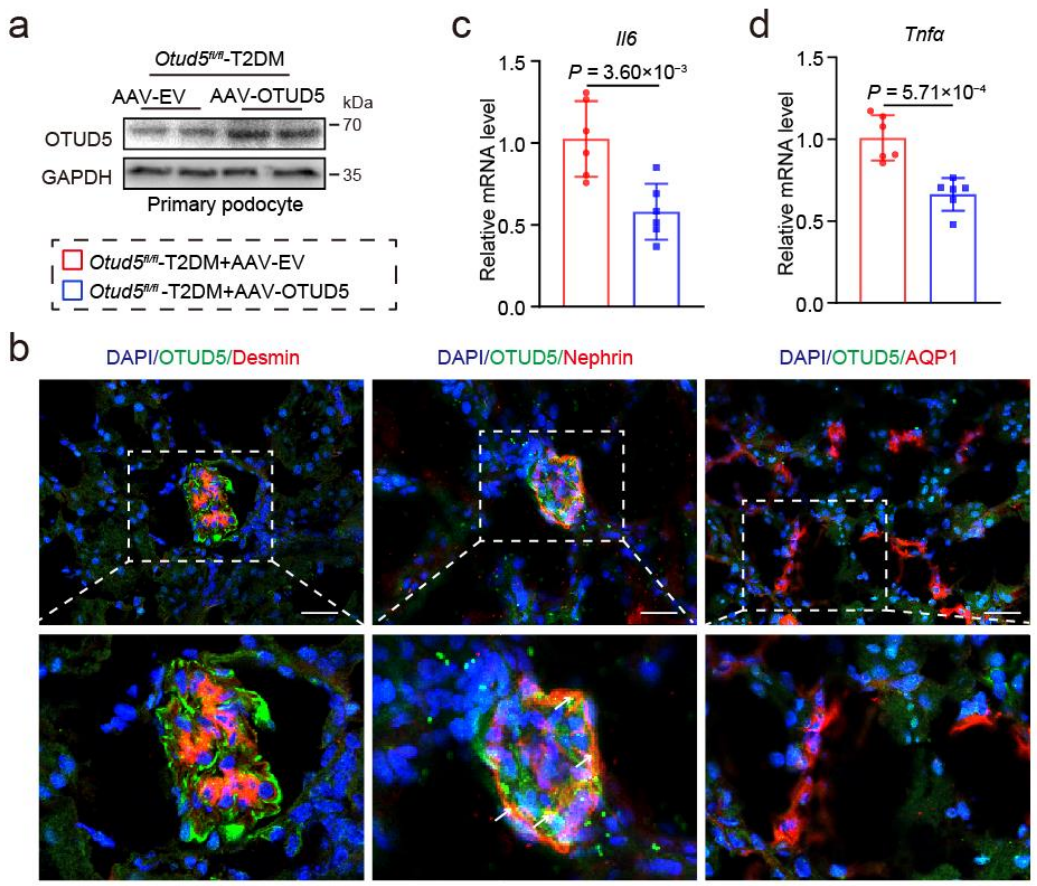

Supplementary Figure 7: Podocyte-specific overexpression of OTUD5 alleviates podocyte inflammatory in T2DM mice.

(a) Representative Western blot of OTUD5 expression in the kidney from *OTUD5$^{fl/fl}$* +AAV-EV and *OTUD5$^{fl/fl}$* +AAV-OTUD5 mice induced by T2DM. (*n* = 6 samples). (b) Representative immunofluorescence staining images of kidney tissues from *OTUD5$^{fl/fl}$* +AAV-OTUD5 mice, showing immunoreactivity to OTUD5 (green) and Desmin or Nephrin or AQP1(red). Tissues were counterstained with DAPI (blue). Scale bar, 50μm. (c-d) Real-time qPCR showing mRNA levels of *Il6* (b) and *Tnfα* (c) in the kidney tissues of each group. (*n* = 6 samples; *P* values were determined by two-tailed unpaired t test and data are presented as mean ± SD)
