# Supplementary material for: Podocyte OTUD5 alleviates diabetic kidney disease through deubiquitinating TAK1 and reducing podocyte inflammation and injury
Source: Nat Commun. 2024 Jun 27;15:5441. doi: 10.1038/s41467-024-49854-1 (PMC11211476; doi:10.1038/s41467-024-49854-1)
Supplement: Supplementary file 3 — Reporting Summary [file 41467_2024_49854_MOESM3_ESM.pdf]

nature portfolio

Corresponding author(s): Guang Liang

Last updated by author(s): Jun 6, 2024

Reporting Summary

Nature Portfolio wishes to improve the reproducibility of the work that we publish. This form provides structure for consistency and transparency in reporting. For further information on Nature Portfolio policies, see our Editorial Policies and the Editorial Policy Checklist.

Statistics

For all statistical analyses, confirm that the following items are present in the figure legend, table legend, main text, or Methods section.

n/a	Confirmed	
☐	☒	The exact sample size (n) for each experimental group/condition, given as a discrete number and unit of measurement
☐	☒	A statement on whether measurements were taken from distinct samples or whether the same sample was measured repeatedly
☐	☒	The statistical test(s) used AND whether they are one- or two-sided<br>*Only common tests should be described solely by name; describe more complex techniques in the Methods section.*
☒	☐	A description of all covariates tested
☐	☒	A description of any assumptions or corrections, such as tests of normality and adjustment for multiple comparisons
☐	☒	A full description of the statistical parameters including central tendency (e.g. means) or other basic estimates (e.g. regression coefficient) AND variation (e.g. standard deviation) or associated estimates of uncertainty (e.g. confidence intervals)
☐	☒	For null hypothesis testing, the test statistic (e.g. F, t, r) with confidence intervals, effect sizes, degrees of freedom and P value noted<br>*Give P values as exact values whenever suitable.*
☒	☐	For Bayesian analysis, information on the choice of priors and Markov chain Monte Carlo settings
☒	☐	For hierarchical and complex designs, identification of the appropriate level for tests and full reporting of outcomes
☒	☐	Estimates of effect sizes (e.g. Cohen's d, Pearson's r), indicating how they were calculated

Our web collection on statistics for biologists contains articles on many of the points above.

Software and code

Policy information about availability of computer code

Data collection	Blood glucose levels were measured and recorded using a glucometer (One Touch Ultra Easy, Life Scan, USA). Ultrathin sections were examined under a Philips CM 120 electron microscope (Philips Medical Systems, Inc.). The quantitative PCR was performed in a Real-Time PCR System (QuantStudioTM 3, Thermo Fisher Scientific, CA, USA).
Data analysis	Image J analysis software version 1.38e (NIH, Bethesda, MD, USA); GraphPad Prism 8.0 (GraphPad, San Diego, CA)

For manuscripts utilizing custom algorithms or software that are central to the research but not yet described in published literature, software must be made available to editors and reviewers. We strongly encourage code deposition in a community repository (e.g. GitHub). See the Nature Portfolio guidelines for submitting code & software for further information.

Data

Policy information about availability of data

All manuscripts must include a data availability statement. This statement should provide the following information, where applicable:

- Accession codes, unique identifiers, or web links for publicly available datasets
- A description of any restrictions on data availability
- For clinical datasets or third party data, please ensure that the statement adheres to our policy

All data supporting the finding in the study are included in the main article and associated files. Source Data are provided with this manuscript.

Research involving human participants, their data, or biological material

Policy information about studies with human participants or human data. See also policy information about sex, gender (identity/presentation), and sexual orientation and race, ethnicity and racism.

Reporting on sex and gender	We did not actively select the sex or gender of prospectively enrolled patients
Reporting on race, ethnicity, or other socially relevant groupings	Renal biopsies from 3 diabetic patients and 3 nondiabetic patients were collected from the Department of Pathology, the First Affiliated Hospital of Wenzhou Medical University. Participants were asked to identify their gender, which was recorded as male or female.
Population characteristics	Our human study does not quality as a clinical trial according to the NIH clinical trial definition. The clinical characteristics of the patients are reported in Supplementary Table 2.
Recruitment	Renal biopsies from 3 diabetic patients and 3 nondiabetic patients were collected from the Department of Pathology, the First Affiliated Hospital of Wenzhou Medical University. The nondiabetic samples were obtained from the healthy kidney poles of individuals who underwent tumor nephrectomies without diabetes or renal disease.
Ethics oversight	All experiments involving human samples were approved by the Ethics Committee in Clinical Research of the First Affiliated Hospital of Wenzhou Medical University (Wenzhou, China; Approval number: 2023-0115), and informed consent was obtained from the patients. All aspects of the study followed the Declaration of Helsinki of 1975, revised in 2008.

Note that full information on the approval of the study protocol must also be provided in the manuscript.

Field-specific reporting

Please select the one below that is the best fit for your research. If you are not sure, read the appropriate sections before making your selection.

☒ Life sciences ☐ Behavioural & social sciences ☐ Ecological, evolutionary & environmental sciences

For a reference copy of the document with all sections, see nature.com/documents/nr-reporting-summary-flat.pdf

Life sciences study design

All studies must disclose on these points even when the disclosure is negative.

Sample size	For in vitro experiments, no statistical methods were used to pre-determine sample size but our sample sizes are similar to those reported in previous publications (doi: 10.1161/CIRCRESAHA.119.315861, doi: 10.1038/s41467-020-15592-3). For mouse experiments, the sample sizes were defined by a prior power calculation with G-Power 3.1.9 software (http://www.gpower.hhu.de/), considering a statistical power of 80% and α=0.05
Data exclusions	No data were excluded from the analysis.
Replication	The experiments were independently repeated multiple times, and statistical tests have been performed to ensure reproducibility. Information on statistical tests and reproducibility are described in the figure legends.
Randomization	Sample groups were allocated randomly.
Blinding	In all experiments, investigators were blinded to group identification during data collection and processing.

Reporting for specific materials, systems and methods

We require information from authors about some types of materials, experimental systems and methods used in many studies. Here, indicate whether each material, system or method listed is relevant to your study. If you are not sure if a list item applies to your research, read the appropriate section before selecting a response.

Materials & experimental systems

n/a	Involved in the study	
☐	☒	Antibodies
☐	☒	Eukaryotic cell lines
☒	☐	Palaeontology and archaeology
☐	☒	Animals and other organisms
☒	☐	Clinical data
☒	☐	Dual use research of concern
☒	☐	Plants

Methods

n/a	Involved in the study	
☒	☐	ChIP-seq
☒	☐	Flow cytometry
☒	☐	MRI-based neuroimaging

Antibodies

Antibodies used	Antibodies against OTUD5 (#21002-1-AP), FLAG-Tag (#20543-1-AP), HA-Tag (#51064-2-AP), His-Tag (#66005-1-IG), Rabbit IgG (#30000-0-AP), Mouse IgG (#B900620) were purchased from Proteintech (Wuhan, China). Antibodies against ERK (#sc-514302), P-ERK (#sc-7383), JNK (#sc-7345), and P-JNK (#sc-6254) were purchased from Santa Cruz (CA, USA). Antibodies against TAK1 (#5206S), P-TAK1 (Ser412) (#9339S), TAB2 (#3745S), Ubiquitin (P4D1) (#3936), P38 (#8690S) and P-P38 (#4631S) were purchased from Cell Signaling Technology (MA, USA). Antibodies against GAPDH (#MB001) were purchased from Bioworld (Missouri, USA).
Validation	Antibodies were previously validated for immunoblot or immunofluorescence of human or mouse sample either in the laboratories of ourselves or commercial suppliers, see below for details:<br>Anti-OTUD5 (1002-1-AP) has been quality validated in immunoblot and immunofluorescence in human sample by previous study (doi: 10.7554/eLife.81247) and also stated in the manufacture's website.<br>Anti-Flag (20543-1-AP) has been quality validated in immunoblot in mouse sample by previous study (doi: 10.1161/CIRCRESAHA.122.321849) and also stated in the manufacture's website.<br>Anti-HA (51064-2-AP) has been quality validated in immunoblot in mouse sample, as stated in the manufacture's website.<br>Anti-His (6005-1-IG) has been quality validated in immunoblot in mouse or human sample by previous study (doi: 10.1053/j.gastro.2022.11.025) and also stated in the manufacture's website.<br>Anti-ERK (sc-514302) has been quality validated in immunoblot in mouse sample by previous study (doi: 10.1186/s12943-023-01817-8) and also stated in the manufacture's website.<br>Anti-P-ERK (sc-7383) has been quality validated in immunoblot in mouse sample by previous study (doi: 10.1038/s41467-023-38430-8) and also stated in the manufacture's website.<br>Anti-JNK (sc-7345) has been quality validated in immunoblot in mouse sample by previous study (doi: 10.1084/jem.20201763) and also stated in the manufacture's website.<br>Anti-P-JNK (sc-6254) has been quality validated in immunoblot in mouse sample by previous study (doi: 10.1016/j.molcel.2023.07.019) and also stated in the manufacture's website.<br>Anti-TAK1 (5206S) has been quality validated in immunoblot in mouse sample by previous study (doi: 10.1038/s41467-022-33862-0) and also stated in the manufacture's website.<br>Anti-P-TAK1 (9339S) has been quality validated in immunoblot in mouse sample by previous study (doi: 10.1038/s41467-023-36154-3) and also stated in the manufacture's website.<br>Anti-TAB2 (3745S) has been quality validated in immunoblot in mouse sample by previous study (doi: 10.1038/s41467-023-36625-7) and also stated in the manufacture's website.<br>Anti-Ubiquitin (3936) has been quality validated in immunoblot in mouse sample by previous study (doi: 10.1038/s41467-023-42829-8) and also stated in the manufacture's website.<br>Anti-P38 (8690S) has been quality validated in immunoblot in mouse sample by previous study (doi: 10.1038/s41467-023-43369-x) and also stated in the manufacture's website.<br>Anti-P-P38 (4631S) has been quality validated in immunoblot in mouse or human sample by previous study (doi: 10.7150/thno.74753) and also stated in the manufacture's website.<br>Anti-GAPDH (MB001) has been quality validated in mouse sample, as stated in the manufacture's website.

Eukaryotic cell lines

Policy information about cell lines and Sex and Gender in Research

Cell line source(s)	The mice renal podocyte cell line (MPC5) was obtained from the Cell Bank of the Chinese Academy of Sciences (Shanghai, China). NIH/3T3 cell line was obtained from the Shanghai Institute of Biochemistry and Cell Biology (Shanghai, China)
Authentication	MPC5 and NIH/3T3 cell lines were authenticated by PCR assays with species-specific primer.
Mycoplasma contamination	Cell lines were tested to be mycoplasma-negative by the standard PCR method.
Commonly misidentified lines (See ICLAC register)	No commonly misidentified cell line was used.

Animals and other research organisms

Policy information about studies involving animals; ARRIVE guidelines recommended for reporting animal research, and Sex and Gender in Research

Laboratory animals	Podocyte-specific Otud5 knockout Mice (Nphs1-Cre OTUD5fl/fl mice; OTUD5CKO) were obtained from Gempharmatech Co., Ltd (Jiangsu, China). The genotype of OTUD5CKO was maintained by crossing the C57BL/6JGpt-Otud5em1Cflox/Gpt mouse (OTUD5fl/fl, strain No. T052115) and the C57BL/6JGpt-H11em1Cin(Nphs1-iCre)/Gpt mouse (Myh6-iCre, strain No. T005680). The mice were housed in an environmentally controlled room at 22 ± 2.0 °C and 50% ± 5% humidity, with a 12-hour light/dark cycle, and were fed standard rodent chow and tap water. Six weeks old OTUD5CKO and OTUD5f/f mice were used.
Wild animals	The study did not involve wild animals.
Reporting on sex	Male OTUD5CKO and OTUD5f/f mice were used.

Field-collected samples	Study did not involve samples collected from the field.
Ethics oversight	All animal care and handling procedures were conducted according to the National Institutes of Health (USA) guidelines and approved by the Wenzhou Medical University Animal Policy and Welfare Committee (Approval number: Wydw2021-0182).

Note that full information on the approval of the study protocol must also be provided in the manuscript.

Plants

Seed stocks	*Report on the source of all seed stocks or other plant material used. If applicable, state the seed stock centre and catalogue number. If plant specimens were collected from the field, describe the collection location, date and sampling procedures.*
Novel plant genotypes	*Describe the methods by which all novel plant genotypes were produced. This includes those generated by transgenic approaches, gene editing, chemical/radiation-based mutagenesis and hybridization. For transgenic lines, describe the transformation method, the number of independent lines analyzed and the generation upon which experiments were performed. For gene-edited lines, describe the editor used, the endogenous sequence targeted for editing, the targeting guide RNA sequence (if applicable) and how the editor was applied.*
Authentication	*Describe any authentication procedures for each seed stock used or novel genotype generated. Describe any experiments used to assess the effect of a mutation and, where applicable, how potential secondary effects (e.g. second site T-DNA insertions, mosiacism, off-target gene editing) were examined.*
